# Predicting optimum crop designs using crop models and seasonal climate forecasts

**DOI:** 10.1038/s41598-018-20628-2

**Published:** 2018-02-02

**Authors:** D. Rodriguez, P. de Voil, D. Hudson, J. N. Brown, P. Hayman, H. Marrou, H. Meinke

**Affiliations:** 1 Queensland Alliance for Agriculture and Food Innovation (QAAFI), The University of Queensland, PO Box 102, Toowoomba, Queensland 4350 Australia; 2 Bureau of Meteorology, PO Box 1289, Melbourne, Victoria 3008 Australia; 3 CSIRO Agriculture and Food, 15 College Rd., Sandy Bay, Tasmania 7005 Australia; 4 South Australian Research and Development Institute, PO Box 397, Adelaide, South Australia 5066 Australia; 5 SupAgro, 2 place Viala, 34060, Montpellier, cedex 02 France; 6 University of Tasmania, and Tasmanian Institute of Agriculture, Private Bag 98, Hobart, Tasmania 7001 Australia

## Abstract

Expected increases in food demand and the need to limit the incorporation of new lands into agriculture to curtail emissions, highlight the urgency to bridge productivity gaps, increase farmers profits and manage risks in dryland cropping. A way to bridge those gaps is to identify optimum combination of genetics (G), and agronomic managements (M) i.e. crop designs (GxM), for the prevailing and expected growing environment (E). Our understanding of crop stress physiology indicates that in hindsight, those optimum crop designs should be known, while the main problem is to predict relevant attributes of the E, at the time of sowing, so that optimum GxM combinations could be informed. Here we test our capacity to inform that “hindsight”, by linking a tested crop model (APSIM) with a skillful seasonal climate forecasting system, to answer “What is the value of the skill in seasonal climate forecasting, to inform crop designs?” Results showed that the GCM POAMA-2 was reliable and skillful, and that when linked with APSIM, optimum crop designs could be informed. We conclude that reliable and skillful GCMs that are easily interfaced with crop simulation models, can be used to inform optimum crop designs, increase farmers profits and reduce risks.

## Introduction

Feeding the projected 9 billion people by 2050 will primarily depend on our capacity to increase food production per unit of land area, particularly in dryland cropping^1^, rather than incorporating new lands into agriculture^2,3^. Opportunities to increase yields in dryland cropping systems exist^4,5^, and are likely to contribute up to ca. 46% of the projected future food supply/demand gap^1^. Dryland cropping is typically characterized by varying levels of climate and soils variability that generate different frequencies, dynamics and intensities of water stress patterns and crop yields^6,7^. Amongst most broad acre dryland crops, sorghum is considered to present advantages in terms of heat and drought tolerance^8^, while recent progress in sorghum yield gains^9^ show potential for sorghum to contribute significantly to bridge expected gaps between food production and demand.

Climate variability contributes to yield gaps in three interrelated ways. (i) directly through crop physiological impacts of water and heat stresses^7^; (ii) indirectly when risk averse farmers adopt conservative management strategies that trade yield potential for reduced yield variability^10^; and (iii) through a ‘moving target effect’ where even risk neutral farmers generally opt for management options that suit an average season, but that are sub-optimal for either above or below average seasons^11^.

In dryland sorghum cropping, growing conditions are influenced by two major factors, namely soil conditions at the time of sowing (i.e. soil fertility and soil water availability) and in-crop rainfall and its distribution during the upcoming season. While soil conditions are usually known (or at least knowable) at the beginning of the season, information about future rainfall could be obtained via skillful probabilistic seasonal climate forecasts^12^. Since the early 1980s there has been a steady improvement in the skill of seasonal climate forecasts^13^. This has come in part from advances in the understanding of climate drivers and sources of predictability at a range of time scales^14^, but also observational and computing advances^13^. Improvements in skill have also been greatly assisted by satellite and computing technology and improvements in data assimilation and model parameterisation^14^. For example, a comparative assessment of accuracy and reliability of Australia’s Global Circulation Model (GCM) operational dynamic forecasting model, versus the previous statistical seasonal forecasting system, showed that the GCM based system was more reliable, consistently more accurate over a larger spatial domain, and more useful than the previous Bureau of Meteorology statistical model^15^.

In agriculture, seasonal climate forecasts are particularly useful when linked with crop simulation models^16,17^. The significance of using seasonal climate forecasts linked with crop simulation tools^7,18,19^ resides in the capacity of dynamic crop models to capture the climate – soil – crop interactions and their emerging dynamics on water and nutrient supply and demand, stress patterns and interactions determining the final yield. Recent improvements in the capacity of the APSIM (www.apsim.info) crop simulation model to model the physiology and genetics of complex adaptive traits in sorghum^20^, provides opportunity to inform optimum combinations of genetics (G) and managements (M), known as ‘crop designs’ (GxM)^7^.

Both, gains in skill from the adoption of dynamic GCM forecasting systems, and improvements in crop simulation capacity^20,21^, provide opportunity to develop new and more valuable climate applications. This calls for revisiting the quantification of value in the skill of existing crop modelling and climate forecasting tools to support crop design decisions and to bridge productivity gaps by providing benefits to dryland farmers world-wide. Here, the research question “What is the value of the skill in seasonal climate forecasting, to inform crop designs?” was answered by

(i) identifying a reliable and skillful seasonal climate forecast;
(ii) understanding our capacity to predict outcomes of alternative crop designs; and
(iii) using a reliable and skillful seasonal climate forecast and a crop model to quantify, in principle, the magnitude of potential changes in profits and risks from adapting crop designs to the ‘where and when’, i.e. location-specific and expected seasonal conditions.

## Results

### Rainfall forecasts reliability and measures of skill

**Figure 1:**
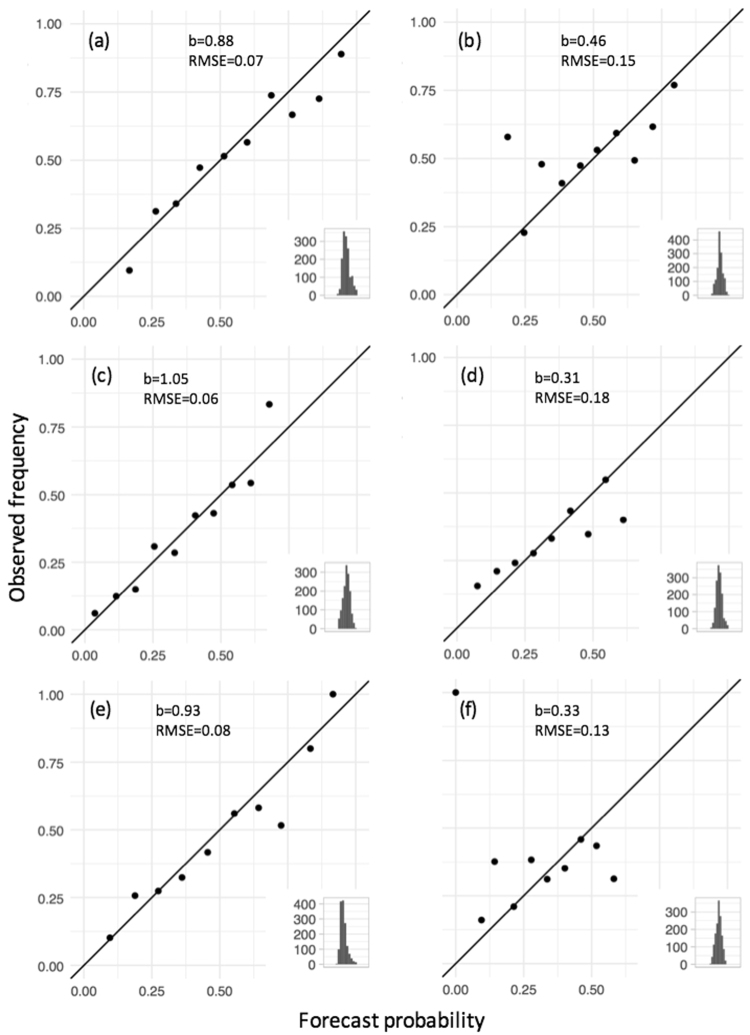
Reliability plots of forecast probabilities for monthly rainfall i.e. lead time = 0, for POAMA2 (**a**,**c**, and **e**) and the SOI phase system (**b**,**d**, and **f**), for above and below long-term median (**a** and **b**), and rainfall falling in the first (**c** and **d**), and third (**e** and **f**) terciles, at Moree, Goondiwindi, Dalby and Emerald Australia. In each graph, the solid line is the 1:1 relationship; *b* is the slope of the relationship between the observed frequency and forecasted probability; and RMSE is the root mean square error for the regression.

The skill of two forecast systems – the SOI phase system and the GCM POAMA-2 (see Supp. Mat.) – were compared relative to climatology. Reliability plots of three-month rainfall forecast probabilities at a lead time = 0, for above median rainfall (Fig. 1a,b), and rainfall falling in the first (Fig. 1c,d) and third (Fig. 1e,f) terciles, showed that POAMA-2 had clear advantages over the statistical SOI phase system, in terms of reduced biases, higher reliability and sharpness. The reliability for above/below median rainfall, tercile 1 and 3 rainfall were always higher for POAMA-2 i.e. in Fig. 1, *b* (slope) values were much closer to the unity, and root mean square error (RMSE) values were less than half of those for the SOI phase system. The frequency plots in the insets of Fig. 1, represent the relative fraction of grid points occurring for each forecast probability bin, and indicate a relatively sharper forecast (a greater range of probabilities issued) with POAMA-2, making it potentially more useful for decision making i.e. an improved capacity to predict extreme seasons.

**Figure 2:**
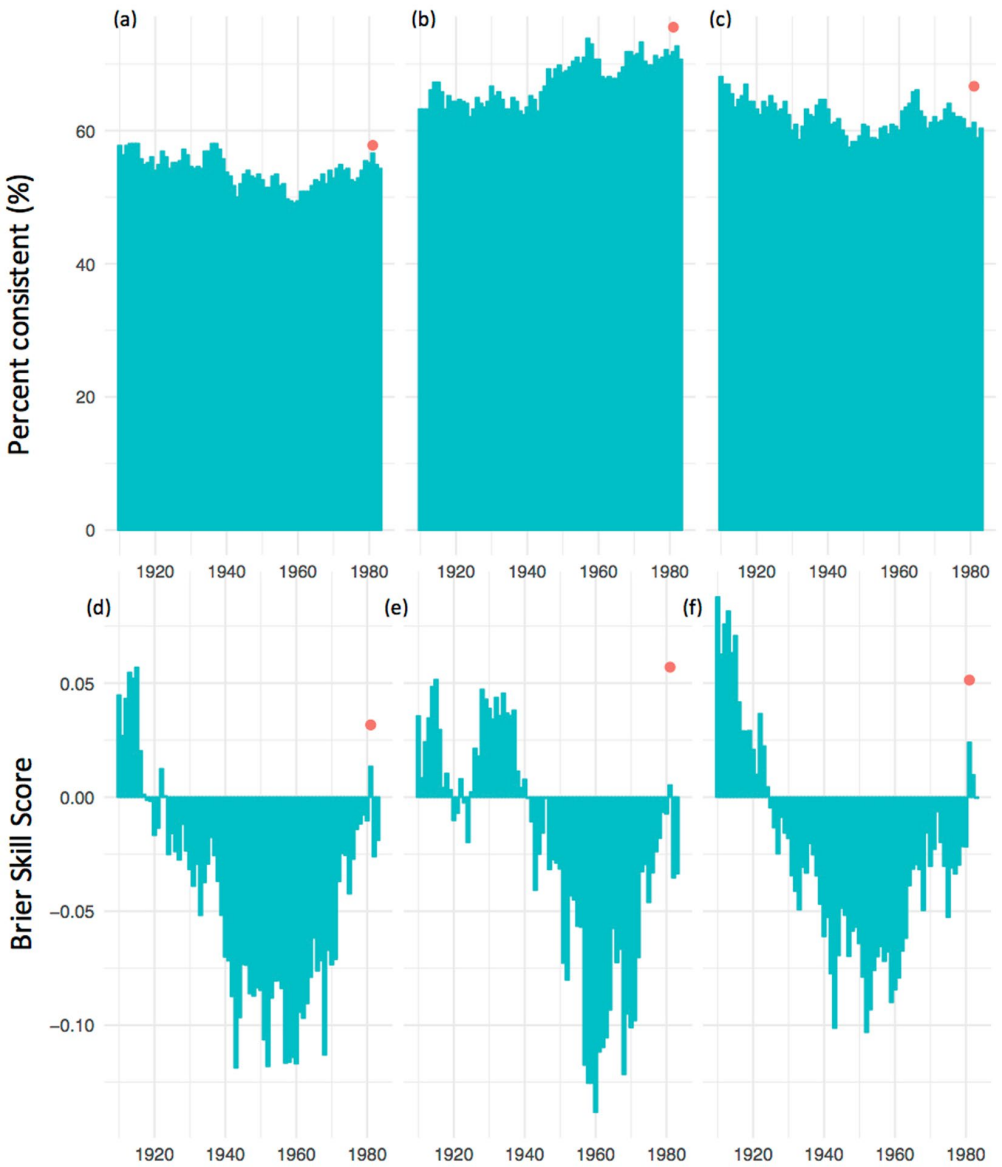
Thirty year running percent consistent (**a** to **c**), and Brier Skill Score (**d** to **f**) (Murphy, 1986) for the SOI phase system from 1920 to 1983 for forecasts of above and below median rainfall (**a** and **d**), rainfall falling on tercile 1 (**b** and **e**), and rainfall falling tercile 3 (**c** and **f**). Forecasts are for September to November rainfall, all sites i.e. Moree, Goondiwindi, Dalby and Emerald Australia together. The red dot is the skill for POAMA-2 for the period 1981–2013.

**Figure 3:**
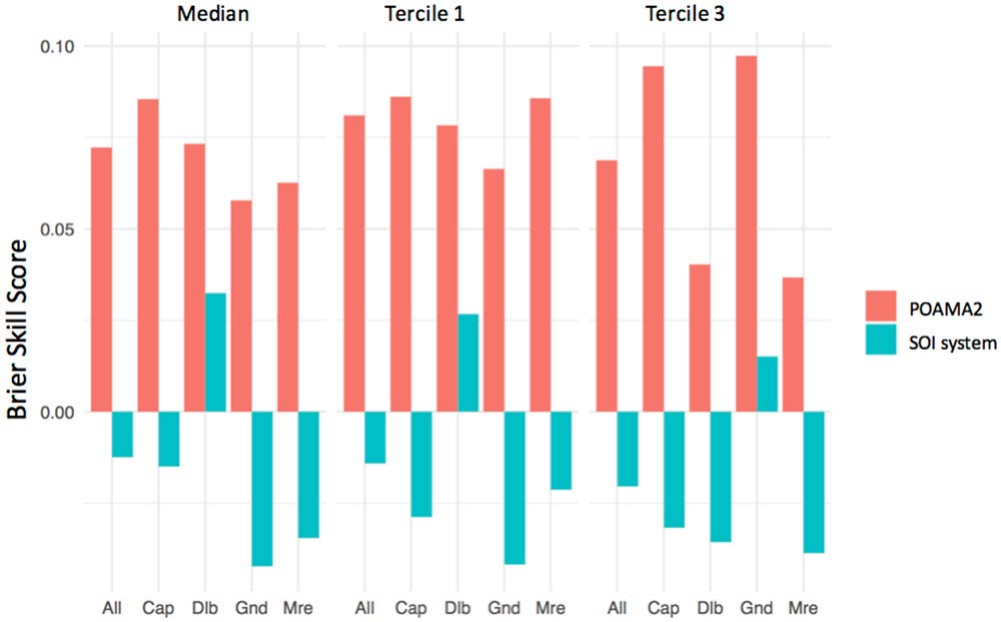
Brier Skill Score for above and below median, tercile 1 and tercile 3 three monthly rainfall (lead time = 0) from January to December, using POAMA-2 and the SOI phase system, at all three locations (All), Capella (Cap), Dalby (Dalb), Goondiwindi (Gnd), and Moree (Mre) Australia.

**Figure 4:**
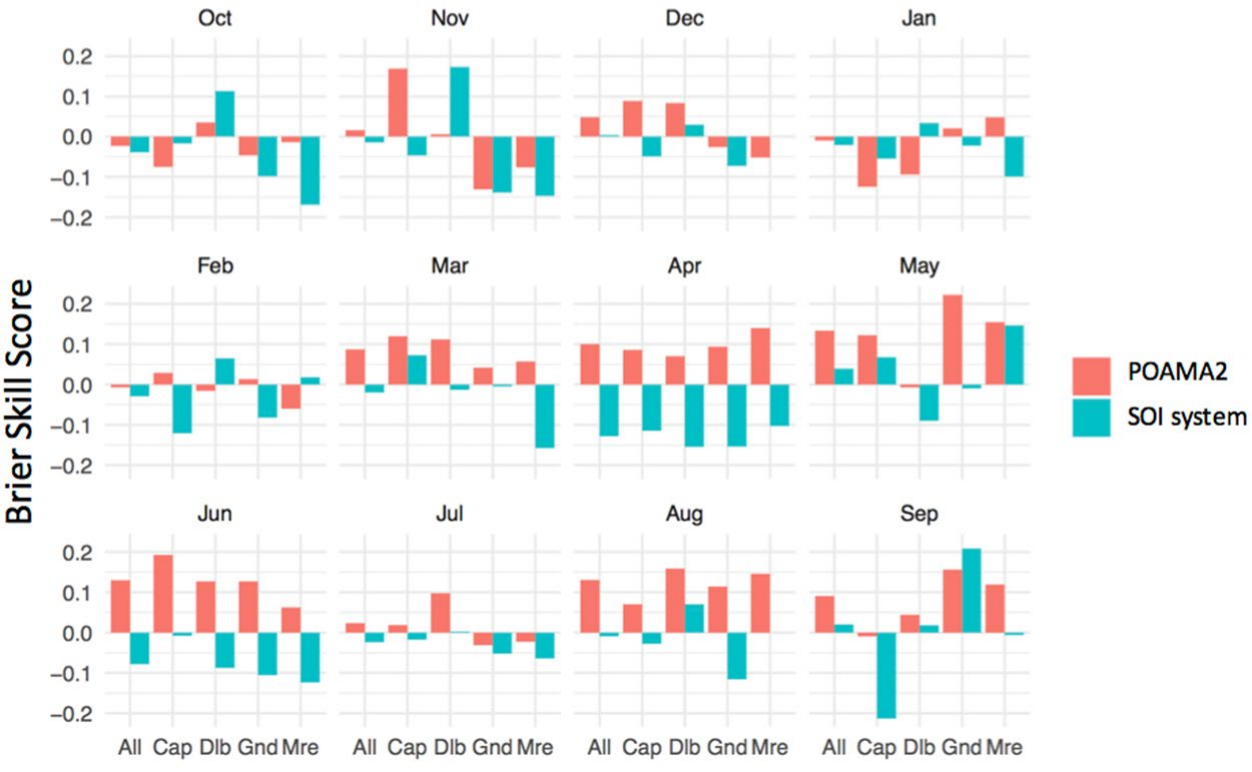
Brier Skill Scores for forecasts of above/below median three-monthly rainfall forecasted using POAMA2 and the SOI phase system for: all three locations (All), and for Capella (Cap), Dalby (Dalb), Goondiwindi (Gnd), and Moree (Mre) Australia. The month indicated on the figure corresponds to the first month of the forecast (i.e. for “Oct” the forecast is initialized on the 1st Oct and is assessed for the OND season).

Thirty-year-running percent-consistent forecasts and Brier skill score (BSS) values indicate the changing and lower skill level of the SOI phase system compared to the skill of POAMA-2 over the hindcast period 1981–2013 (Fig. 2). We also present spatial (Figs 3, 4) and seasonal (Fig. 4) variations in BSS for above/below median rainfall forecasts. In contrast to the SOI phase system, Fig. 3 shows that overall and for all the sites, the forecasts from POAMA-2 were more skillful than using climatology. For the forecasts of different seasons, POAMA-2 showed consistently higher values of BSS for above/below median rainfall forecasts across most sites compared to the SOI phase system, particularly for forecasts initialized between March and September, when POAMA-2 has its highest skill (Fig. 4). We found similar results for forecasts of rainfall falling within terciles 1 and 3 of climatology (Figures S1 and S2).

**Figure 5:**
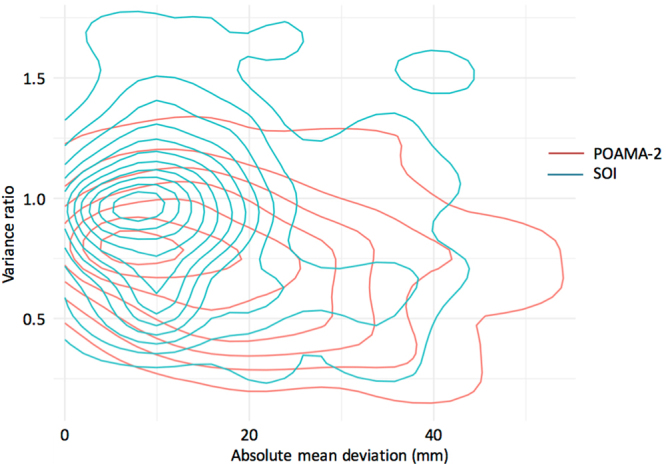
Relationship between the variance ratio i.e. a measure of dispersion, and the absolute mean deviation (mm) i.e. a measure of shift, for forecasts of above median three-monthly rainfall using POAMA2 and the SOI phase system. The figure is for all months and locations in the study i.e. Capella, Dalby, and Goondiwindi, and Moree Australia.

Changes in shift and dispersion between climatology and the forecasted distributions for three monthly above/ below median rainfall are shown in density plots, as the relationship between the variance ratio (VR) and the absolute mean deviations (AMD) (mm) (Fig. 5 all months and locations, and Fig. S3 for each monthly forecast of three-monthly rainfall). Figure 5 shows a larger frequency of lower VR and higher AMD values for POAMA-2 than for the SOI phase system. Also, POAMA-2 consistently showed lower VR and higher AMD values for rainfall forecasts of each month of the year (Fig. S3).

Given the better performance of POAMA-2 with respect to the SOI phase system, GxExM effects and the value of the forecast skill to inform crop design was only tested using POAMA-2.

**Table 1:** Rainfall characteristics of the locations in the study region.

Location	SILO station id.	Longitude	Latitude	Annual rainfall (mm)	CV	Sep-Feb rainfall (mm)	CV
Capella, Queensland	35016	148.0	−23.1	596.1	0.34	397.1	0.40
Dalby, Queensland	41023	151.3	−27.2	669.8	0.25	426.2	0.32
Goondiwindi, Queensland	41038	150.3	−28.6	614.0	0.27	366.9	0.35
Moree, New South Wales	53027	149.9	−29.5	584.3	0.28	339.1	0.38

CV is the coefficient of variability for annual rainfall and September to February rainfall.

### GxExM effects on forecasts of sorghum yield

**Figure 6:**
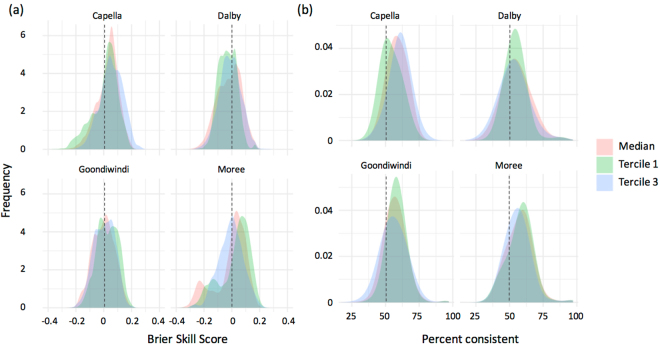
Brier Skill Scores (**a**) and Percent consistent values (**b**) for forecasts of above or below median, tercile 1, and tercile 3 sorghum yields simulated using a factorial combination of genetic, management and site conditions, using POAMA- at Capella, Dalby, and Goondiwindi, and Moree Australia.

We used POAMA-2 ensemble seasonal forecasts downscaled to station level for the period 1981–2015 to drive multiple sets of APSIM simulations and produce simulated sorghum yields. The sets of APSIM simulations covered each possible combination of location; hybrid characteristics (i.e. maturity and tillering habit); management practices (i.e. sowing window, sowing density, row configuration, and level of nitrogen application); location (i.e. soil type); and prevailing conditions (i.e. soil water content at the time of sowing) (Table 2). The BSS and percent consistent values were calculated for sorghum yields and the distributions of BSS and percent consistent values for forecasts of above/below median, tercile 1 and tercile 3 sorghum yields are shown in Fig. 6a,b. The frequency distribution of positive (skillful) BSS values, and values of percent consistent higher than 50% varied across locations and forecasts. An analysis of variance on the values of BSS for the locations and factors in the GxExM factorial combination showed that the factor ‘Location’ had a highly significant (p < 0.0001) effect on the BSS (Table 3, and S1). The influence of the different factors in the GxM factorial was then tested within each location. Results showed that with the exception of soil type and sowing density at Dalby, and row configuration and nitrogen fertilization at Goondiwindi and Moree, most factors significantly affected the value of BSS (Table 3, and S1).

**Table 2:** Location and soil denominator, soil type, and soil water characteristics at the locations in the study region.

Location	Soil type	PAW (mm)	PAWC (mm)
Capella, high	Black clay Vertosol	287	415
Capella, medium	Grey medium clay Vertosol	189	274
Capella, low	Black light clay Vertosol	146	146
Dalby, high	Black clay Vertosol	308	400
Dalby, medium	Black medium clay Vertosol	253	344
Dalby, low	Grey light clay Vertosol	175	274
Goondiwindi, high	Grey clay Vertosol	283	313
Goondiwindi, medium	Grey medium clay Vertosol	214	361
Goondiwindi, low	Grey light clay Vertosol	179	310
Moree, high	Black clay Vertosol	299	254
Moree, medium	Grey medium clay Vertosol	220	304
Moree, low	Black light clay Vertosol	106	124

Plant available water (PAW) is the difference between the drainage upper limit and the crop lower limit; Plant available water capacity (PAWC) is the difference between the drainage upper limit and wilting point.

**Table 3:** Levels of significance from ANOVA for variables related to genotype characteristics (G), management practices (M) and location (E), for all the locations together (All sites); and Capella, Dalby, Goondiwindi, and Moree individually, for the GxMxE combinations showing Brier Skill Scores in the upper 75% of the values.

	All sites	Capella	Dalby	Goondiwindi	Moore
Location	***	na	na	na	na
Sowing window	***	***	***	***	***
Soil type	***	***	ns	***	***
Plant available water	***	***	***	***	***
Hybrid maturity	***	***	***	***	***
Tillering type	***	***	**	*	*
Density	***	***	ns	***	***
Row configuration	***	**	***	ns	ns
Nitrogen fertilization	***	***	***	ns	ns

Significance codes: ***0; **0.001; **0.01; and *0.05; ns = not significant.

na = not applicable.

**Figure 7:**
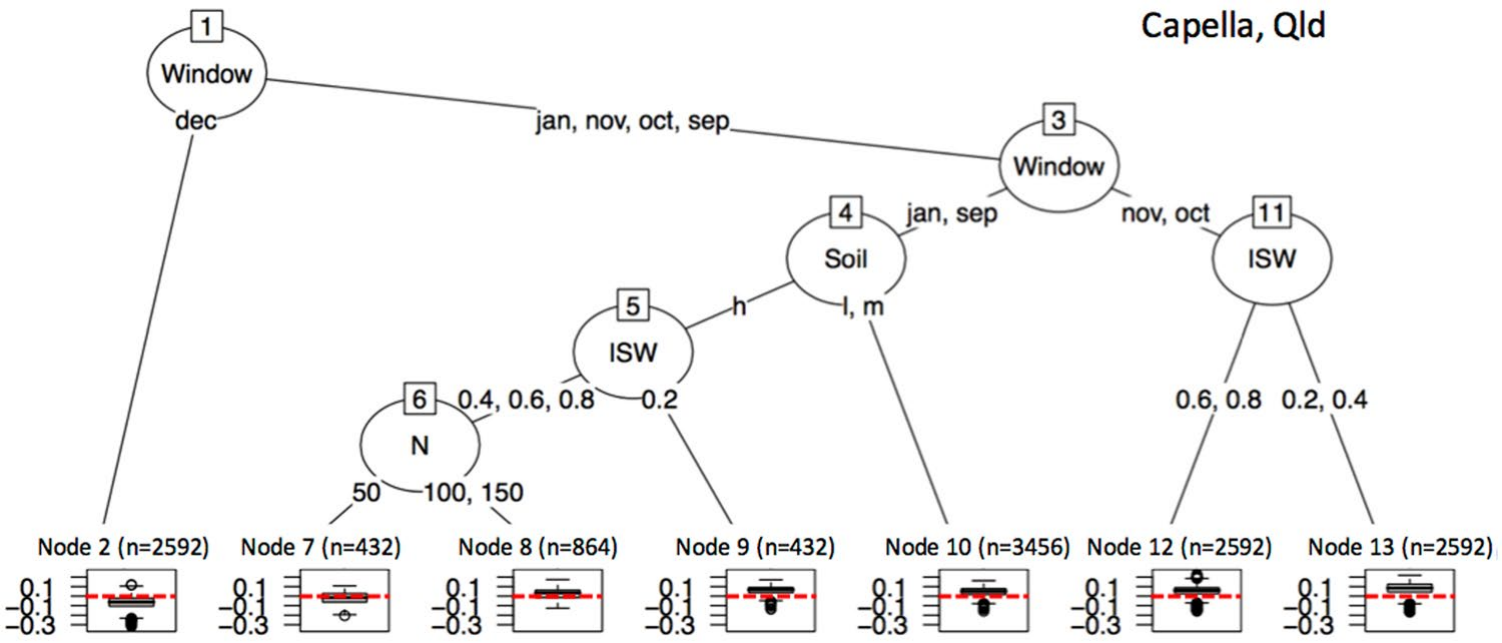
Regression tree on the Brier Skill Scores for forecasts of above median sorghum yields simulated using a factorial combination of genetic, management and site conditions, using POAMA-2 at Capella Australia. References: Window indicates the sowing month; Soil refers to the soil high (h), medium (m) or low (l) plant available water capacity; ISW refers to the soil plant available water content (v v^−1^) at the time of sowing; and N to kg N ha^−1^ applied at sowing. In the boxplots n refers to the number of node simulations and the red dashed line indicates a Brier Skill Score value of 0.

**Figure 8:**
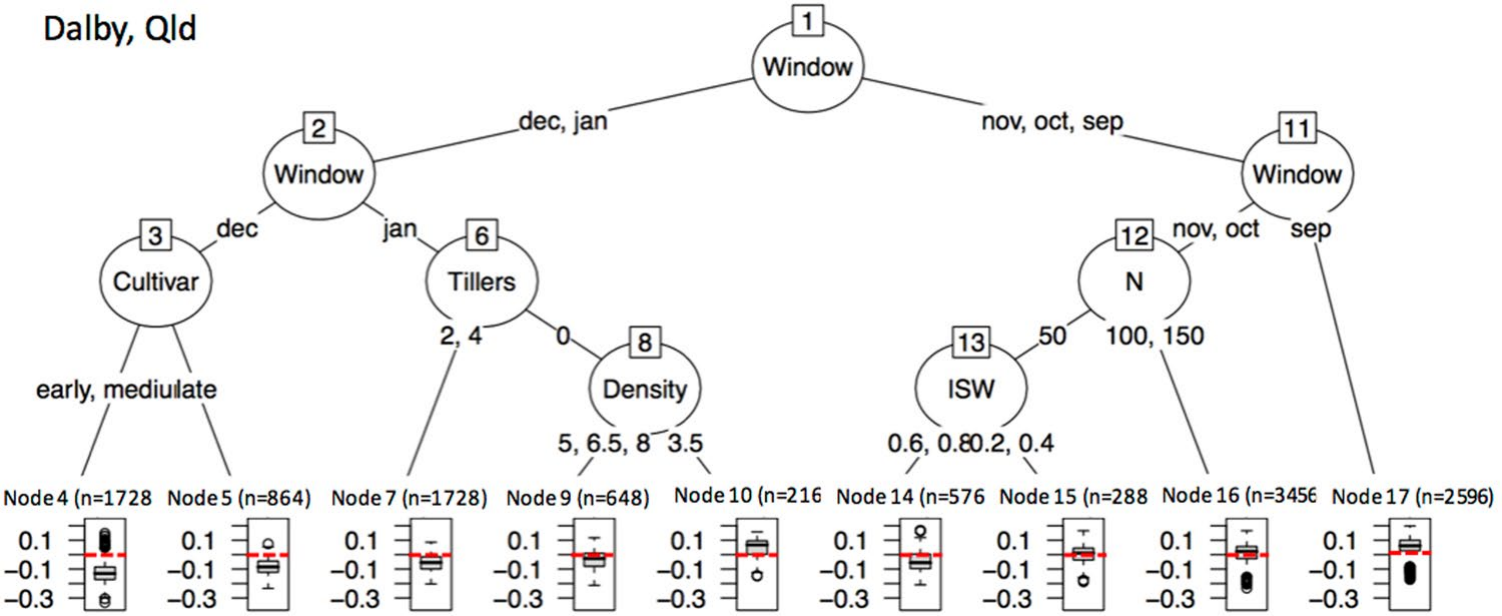
Regression tree on the Brier Skill Scores for forecasts of above or below median sorghum yields simulated using a factorial combination of genetic, management and site conditions, using POAMA2 at Dalby Australia. References: Window indicates the sowing month; Tillers refers to the tillering type of the hybrid where 0 is no tillering and 4 is high tillering; ISW refers to the soil plant available water content (v v^−1^) at the time of sowing; Density to the number of plants sown m^−2^; Cultivar is the maturity type of the hybrid, i.e. early, medium, or late; and N to kg N ha^−1^ applied at sowing. In the boxplots n refers to the number of node simulations and the red dashed line indicates a Brier Skill Score value of 0.

**Figure 9:**
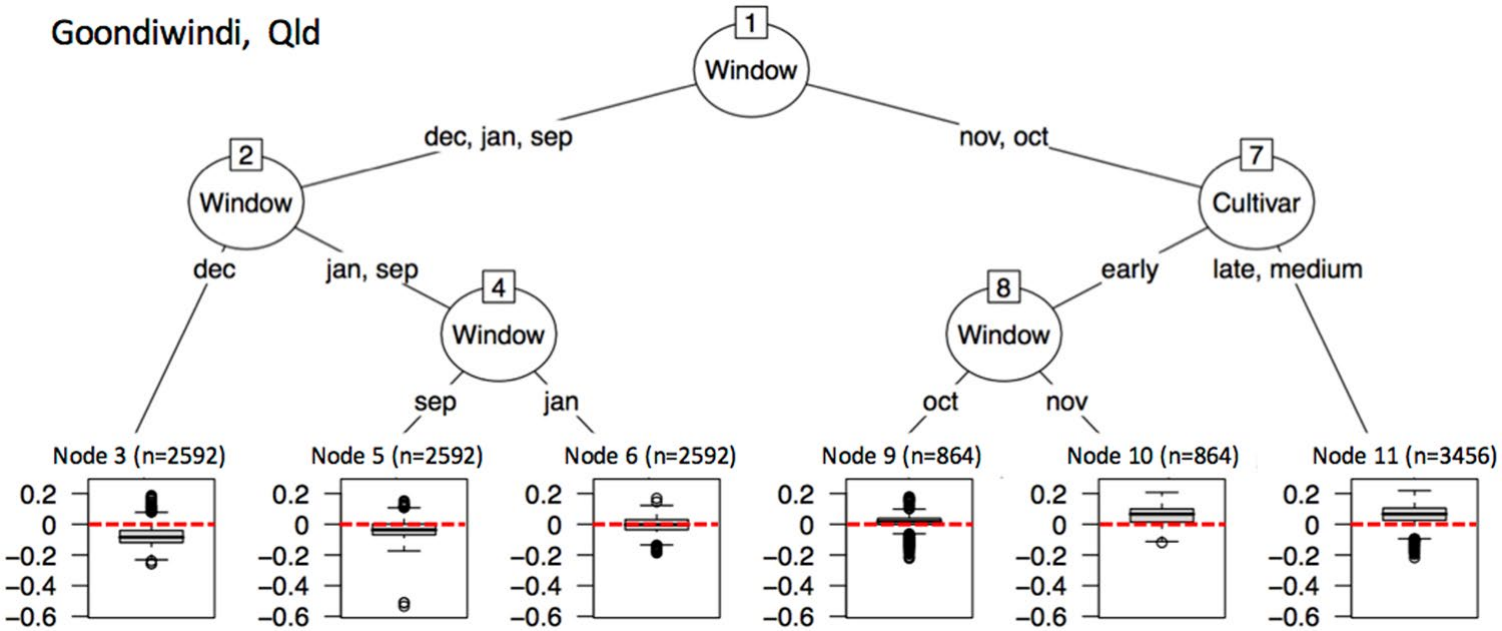
Regression tree on the Brier Skill Scores for forecasts of above or below median sorghum yields simulated using a factorial combination of genetic, management and site conditions, using POAMA2 at Goondiwindi Australia. References: Window indicates the sowing month; and Cultivar is the maturity type of the hybrid i.e. early, medium or late. In the boxplots n refers to the number of node simulations and the red dashed line indicates a Brier Skill Score value of 0.

**Figure 10:**
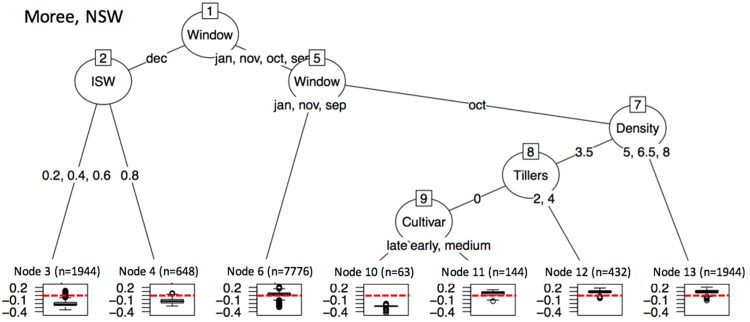
Regression tree on the Brier Skill Scores for forecasts of above or below median sorghum yields simulated using a factorial combination of genetic, management and site conditions, using POAMA2 at Moree Australia. References: Window indicates the sowing month; Tillers refers to the tillering type of the hybrid where 0 is no tillering and 4 is high tillering; ISW refers to the soil plant available water content (v v^−1^) at the time of sowing; Density to the number of plants sown m^−2^; and Cultivar is the maturity type of the hybrid, i.e. early, medium, or late. In the boxplots n refers to the number of node simulations and the red dashed line indicates a Brier Skill Score value of 0.

We used regression trees to untangle the different GxExM effects on the value of BSS (Figs 7–10) and to answer what GxExM combinations show high or low skill values. The different G, E, and M factorial components had different importance in determining positive and high values of BSS i.e. high skill. For example, in Capella Queensland (Fig. 7) the highest values of BSS (high skill) were obtained for sorghum crops sown in October and November (Nodes 12 and 13), particularly on dry soils (Node 13). January and September sowings on high PAWC dry soils (Node 9) also showed high values of the BSS. The lowest values of BSS (no skill) were observed for December sowings (Node 2). In Dalby (Fig. 8) the highest values of BSS (high skill) were obtained for sorghum crops sown in early in September (Node 17), or with late sowings in January, of non-tillering hybrids sown at low plant densities (Node 10). In Dalby, the lowest values of BSS (no skill) were observed for December sowings (Nodes 4 and 5). In Goondiwindi (Fig. 9) the highest values of BSS (high skill) were obtained for medium and late sorghum crops sown in October and November (Node 11) or early hybrids sown in November (Node 10). In Goondiwindi, the lowest values of BSS (no skill) were observed for December sowings (Nodes 3). In Moree (Fig. 10) the highest values of BSS (high skill) were obtained for sowings in October and high sowing densities (Node 13), or low sow densities of high tillering hybrids (Node 12). In Moree, the lowest values of BSS (no skill) were observed for December sowings particularly on dry soils (Nodes 3), or October sowings of no tillering late hybrids.

In general, the results from the regression trees across all locations followed a similar pattern: higher values of BSS (high skill) were observed when the GxExM factor combination resulted in crop designs that were highly dependent on seasonal conditions, i.e. in-crop rainfall.

### Value of APSIM and POAMA-2 to inform crop designs (GxM)

The value of linking APSIM and a GCM (POAMA-2) to optimize crop designs for each individual year in the hindcast series requires time series of simulated sorghum yields for three distinct different crop design (GxM) strategies. Reflecting current farmer management (GxM_f_) we used a single and static hybrid by management combination optimized for most years (GxM_optS_); and a dynamic GxM choice optimized for each individual year in response to the seasonal climate forecast (GxM_optSCF_). The farmer GxM_f_ and optimum GxM_optS_ managements do not change during the hindcast period 1981 to 2015. In contrast, GxM_optSCF_ is adjusted each year in response to the climate forecast, equations (1) and (2). At each location, the three-time series of simulated yields were converted to an economic time series of profit ($/ha) by multiplying the yield (t/ha) by a price ($/t) to calculate annual income ($/ha) and subtracting a growing cost ($/ha). The current farmer management (GxM_f_) was used as the base line to calculate the benefit of optimizing the hybrid and management in all years (Tables 4 and S2).

1 $${{\rm{Value}}}_{{\rm{optS}}}={{\rm{Profit}}}_{{{\rm{GxM}}}_{{\rm{optS}}}}-{{\rm{Profit}}}_{{{\rm{GxM}}}_{{\rm{f}}}}$$

2 $${{\rm{Value}}}_{{\rm{optSCF}}}={{\rm{Profit}}}_{{{\rm{GxM}}}_{{\rm{optSCF}}}}-{{\rm{Profit}}}_{{{\rm{GxM}}}_{{\rm{optS}}}}$$

Irrespective of the calculation method, the value of linking APSIM and POAMA-2 was different across locations and soil types. Within each location Value_optS_ was largest for crops grown on deeper soils, both in terms of higher profits and reductions in down side risk. On average, Value_optS_ showed increases in average profits from 10.2 to 26.1% i.e. 56 to 226 AU$ ha^−1^, and average reductions in down side risk of up to 100% i.e. no risk. Table S2 shows the static, most frequent GxM combinations that resulted in the largest increases in profit and the largest reductions in downside risk. For instance, at Dalby common practice is to sow sorghum in October, on at least 60% ISW (i.e. more than 100 mm of stored soil water), using medium maturities and medium tillering hybrids, sown at 5 plants m^−2^ and using medium levels of ca. 50 kg N ha^−1^. However, using APSIM combined with POAMA-2 we can device a more profitable and less risky static GxM combination: the simulations show that on high PAWC soils, increasing plant populations and nitrogen supply would increase profits by up to 16% in 26% of the hindcast years. On medium PAWC soils, gains in profit were 29% in 26% of the years; while on low PAWC soils, gains in profits were 21% in also 26% of the years (Table S2). When we calculated the value of skill in seasonal climate forecasting relative to an improved static management involving higher levels of investment in nitrogen fertilizers, the additional value of the new climate information was smaller, on average 17 AU$ ha^−1^, i.e. Value_optSCF_ in Table 4. In Table 4 we also show the value of perfect knowledge (Value_PK_), this is, the difference between an optimized crop design and the static farmers’ management, calculated using observed climatology. From comparing Value_PK_, and Value_optS_ (Table 4) we can conclude that present value in the skill of seasonal climate forecasting falls approximately mid-way between ‘no skill’ and perfect knowledge of the future climate.

**Table 4:** Mean profits from a simulation using farmers current practice and climatology (Farmers), perfect knowledge (PK) i.e. optimized crop designs using climatology, optimized crop design using POAMA-2 (Optimised); and value i.e. mean changes in profit and down side risk i.e. likelihood of a profit lower than $600 ha^−1^ for, farmers current practice (Value_f_). Optimized crop design using climatology 1981–2015 and (Value_optS_) and a POAMA-2 seasonal climate forecast (Value_optSCF_)).

	Soil type (PAWC)	Mean value to inform GxM with respect to a static management							
		Profit ($ ha^−1^)					DSR (%)		
		Farmers	Optimized	Value_optS_	Value_optSCF_	Value_PK_	Farmers	Optimized	Value_optS_
Capella	High	1108	1260	152	3	335	0	0	No risk
	Medium	748	824	77	3	224	12	0	−100
	Low	544	600	56	4	219	68	62	−9
Dalby									
	High	1127	1337	210	13	353	0	0	No risk
	Medium	1048	1241	194	17	351	0	0	No risk
	Low	795	913	118	12	288	6	3	−50
Goondiwindi									
	High	866	1092	226	16	432	6	0	−100
	Medium	841	1011	170	63	345	3	0	−100
	Low	678	793	115	6	312	34	6	−82
Moree									
	High	1025	1226	202	23	406	0	0	No risk
	Medium	814	962	148	32	370	9	0	−100
	Low	373	427	54	19	210	89	86	−3.3

Value_optS_ = difference in profit ($ ha^−1^) and down side risk (DSR), between simulation of current farmers’ hybrid by management combination (Value_f_) and a static (every year the same) optimized hybrid by management combination.

Value_optSCF_ = difference in profit ($ ha^−1^), between Value_optS_ and the dynamically (every year different) optimized hybrid by management combination informed by the POAMA-2 seasonal climate forecasts.

Value_PK_ = Value of having perfect knowledge i.e. optimum crop design using observed climatology.

## Discussion

Inherent to dryland cropping is a high level of season-to-season and within-season climate variability^14,22^. This, together with different G and M combinations results in highly contrasting stress environments and crop yields^6,7^. Australia has a long track record of valuable developments in climate sciences and applications^23^, such as the development and use of seasonal climate forecasts to inform likely seasonal conditions and practice change^15,23^, see also Climate Kelpie at http://www.climatekelpie.com.au. However, adoption remains low due to, (i) the perceived low value of the existing skill in the information of seasonal climate forecasts^24^; (ii) the complexities associated with the multiple interactions between factors when managing biological systems (i.e. climate, soil and crop interactions, and their effect on the skill and value of crop yield forecasts)^23^; and (iii) the challenge of understanding and communicating probabilistic information^24^, especially by risk averse farm managers and consultants^10^.

Here we used measures of forecast reliability, skill, shift and dispersion to identify a reliable and skillful seasonal climate forecast for rainfall. We then used the most reliable and skillful forecast in combination with a validated crop simulation model to capture the multiple interactions between sorghum hybrid, management and environmental conditions. Finally, we calculated the value of skill in seasonal climate forecasting to inform optimum crop designs for sorghum in Australia.

Results showed that the forecasts produced by Australia’s operational forecasting system POAMA-2^25^ proved to be more reliable, sharper and consistently more accurate than the SOI phase system^26^. The extent of the difference between both systems was substantial; we know of no previous attempts to compare them. Results show that the dynamic climate model outperformed the statistical climate model across all tested indices (Figs 1 and 2). In general, the SOI phase system was worse than climatology at predicting rainfall. This is likely a consequence of progressive skill degradation over time since the system was first developed in the mid 1990s^26^. Being a static, statistical system based on historical relations between the SOI and future rainfall, it is likely to have suffered from degradation due to climate change and low frequency climate fluctuations that have affected the historical climate record since^27^. Significant improvements in sharpness, reliability and accuracy from the previous Bureau of Meteorology (BOM) statistical^28^ to the current BOM’s dynamic models^25^ were reported before^15^. Once challenges such as data initialization and resolution are addressed, dynamical models are logically able to outperform their simpler statistical counterparts for a number of reasons. Firstly, dynamic models can incorporate a number of climate drivers beyond ENSO such as the Indian Ocean Dipole and monsoon variability and their complex interrelationships. Secondly, climate change and its effect on climate drivers are incorporated into dynamical models whereas statistical approaches are limited to analogues from the past^29^. Also, decadal variations in climate interactions require a long historical record for differing phases to be accounted for^30^, particularly the variations in predictive skill across decades^27^. BSS values for above/below median rainfall, and rainfall falling within tercile 1 or 3, varied across sites (Fig. 3) and within sites during the year (Fig. 4). This is consistent with observations of spatial and temporal variations in the influence of ENSO across eastern Australia^14^. An important observation was the significantly higher level of skill in POAMA-2 between March and September across all tested locations. Although the locations in this study are mostly summer rainfall dominant, this is at a time of the year when some of the most profitable crops are grown in the region e.g. wheat and chickpeas.

When we calculated BSS values for a simulated factorial combination of GxM factors on sorghum yields, we observed a large variation in BSS and percent consistent values for forecasts of above/below median, tercile 1 and tercile 3 sorghum yields (Fig. 6a,b). The large variability in BSS values from the GxMxE factorial combination was expected, as the influence of seasonal climate forecasts on sorghum yield is likely to vary with the reliance of the crop design on in-crop rainfall and seasonal conditions^31^. This is consistent with others^32^, who found that sampling variances increase with increasing forecast accuracy, and with decreasing climatological event probability. Our results showed that location, site, hybrid and management practices all significantly affected the value of BSS, and that as indicated above, the highest values of BSS were observed for crop designs that were highly reliant on in-crop rainfall. The capacity of soils to buffer impacts climate variability and to modify the skill and value of a forecast was shown before^31^. The influence of site, hybrid, and management combinations on the economic value of using a crop model and GCM forecasts is discussed below.

Reliability and skill in a seasonal climate forecast is essential, although it does not guarantee that the information has value, or is of sufficient importance to change the behavior of mostly risk averse decision makers such as dryland farmers^10,33^. Previous analyses showed that up to 20% increases in profit and or up to 35% reductions in risk were possible when tactical adjustments to crop nitrogen management were informed using the SOI phase system^17^. More recently, in silico estimates of benefits from environment specific crop designs e.g. associated with increasing plant densities and sowing low tillering sorghum hybrids, ranged between 0.22 and 0.41 t ha^−1^ ca. 56–104 AU$ ha^−1^ at average sorghum prices (254 AU$ t^−1^), together with slight reductions in down side risk^7^. Here we showed that the value, in terms of increases in profits and reductions in downside risks, from identifying optimum GxM combinations using a crop simulation model and a seasonal climate forecast was significant across all tested locations and soil conditions i.e. soil PAWC values (Table 4). The value in skill depended on the baseline for the comparison: When current farmers’ practice was used as the baseline, linking APSIM sorghum and POAMA-2 increased average profits by 143 AU$ ha^−1^ and reduced or even eliminated down side risk. When the baseline for the comparison was the highest yielding, static hybrid-by-management combination (Table 4 and S2), the actual value of the additional climate information was on average 17 AU$ ha^−1^, which compares to the benefits derived from Australia’s sorghum breeding over the last thirty years^9^ i.e. 2.1% per year, or 44 kg ha^−1^ year^−1^. These results indicate that even though the value of the additional climate information might seem small (Value_optSCF_), its magnitude compares well with that derived from much larger and better funded breeding programs. Much larger benefits (Value_optS_) might be realized when using such insights in discussions with farmers on benefits and risk from increasing investments in dryland cropping^10^ to sustainably bridge productivity and profit gaps in dryland cropping i.e. the difference between $${{\rm{Profit}}}_{{{\rm{GxM}}}_{{\rm{f}}}}$$ and $${{\rm{Profit}}}_{{{\rm{GxM}}}_{{\rm{optS}}}}$$ or $${{\rm{Profit}}}_{{{\rm{GxM}}}_{{\rm{optSCF}}}}$$ (Table 4).

Given the chaotic nature of the atmosphere it is impossible to know exactly how it will evolve beyond a few days^34^, requiring the use of probabilistic forecasts and analyses of changes in probability distribution functions, with respect to climatology^12,18,34^. However, given the hypothetical case that we would have perfect knowledge about the future, we also estimated that the present value in seasonal climate forecasting falls mid-way from having perfect knowledge (Value_PK_ in Table 4). Clearly, communicating probabilistic information requires representing the complete distribution of likely outcomes from a change in practice against the counterfactual of ‘no action’, and accepting that pay-offs will be realized long term, rather than every single occasion the forecast is used. This is important, given that the wide range of individual circumstances affects farmers’ levels of risk aversion and investment capacity. The results presented here indicate that improved agronomic practice and increased investments in fertilizers can lift average profits by up to 82%, while climate forecasts information can contribute up to 12%, a value that is on par with plant breeding.

## Conclusions

We conclude that reliable and skillful dynamic GCM models, interfaced with validated crop simulation models, can now be used to inform optimum crop designs to increase farmers’ profits and reduce risks. Australia’s Bureau of Meteorology current POAMA-2 model outperformed the statistical SOI phase system across all tested measures of reliability, skill, shift and dispersion, particularly during autumn and winter. The expected release of BOM’s new higher resolution and more sophisticated ACCESS-S1 seasonal climate forecast system early during 2018 is likely to increase further the value of climate information when linked with crop simulation models like APSIM. To achieve those gains, improvements in downscaling techniques and real-time access to outputs from BOM’s seasonal climate forecasts will be required. Further, efforts need to continue that makes such information available to decision makers in a form that is understandable and useable.

## Methods

### Climate data and seasonal climate forecasting tools

Long term climate records, the SOI phase system^26^ and a hindcast from the operational Australian seasonal climate forecasting system (POAMA-2)^25^ were assembled for four sites in Australia’s northern grains region i.e. Capella, Dalby and Goondiwindi in Queensland, and Moree in New South Wales (Table 1). Weather data included both station data from SILO (1889–2016) (https://www.longpaddock.qld.gov.au/silo/), and gridded (2.5 degree) data (1901–2013) from GPCC (https://www.esrl.noaa.gov/psd/data/gridded/data.gpcc.html). At each location, “0-lead” rainfall forecasts for the next ninety days were made at the beginning of each month using both POAMA-2 and the SOI phase system for the common hindcast period of 1981–2015.

The SOI phase system^26^ was used to develop forecast distributions from analogue years of observed data, a cross validated probability of falling into each category was calculated as in^17^. The SOI phase system uses five fixed phases derived from consecutive monthly values of the SOI normalized with respect to the base period 1887–1989. The five phases of the SOI are rapidly rising, consistently positive, neutral or near zero, rapidly falling and consistently negative. Analog years corresponding to each of the SOI phases were obtained from the Long Paddock website (www.longpaddock.qld.gov.au). The analogue sets used to create the SOI phase distributions were taken from the period 1901–2013.

POAMA-2 is a global ensemble seasonal forecast system, comprising a coupled ocean-atmosphere model and data assimilation systems for the initialization of the ocean, land and atmosphere^25^. The forecasts are initialized with observational data available at the start of the forecast (to provide the best representation of the current state of the climate system) and are then run forward in forecast mode for nine months. Multiple forecasts are run for a given start time, called an ensemble, in order to provide an indication of the uncertainty of the future evolution of the climate system i.e. the likelihood of future conditions is presented as a forecast distribution. The ensemble for a given start time consists of thirty-three forecasts that differ only in their initial conditions. POAMA-2 forecasts are made every 5 days and in order to expand the number of ensemble members, the outputs from three consecutive runs were used to create a 99-member ensemble (this approach is also taken by the Bureau of Meteorology for their climate outlooks). POAMA-2 forecasts were made for four locations each falling into a separate 2.5-degree grid cell. To assess the value of using POAMA-2 seasonal forecasts, we use a set of forecasts that have been run retrospectively over a period in the past, called a hindcast set. The hindcast period is 1981–2015 and the 99-member ensemble forecasts issued on the first day of every month in that period were used in this study. POAMA-2 forecasts were assembled in two forms, (i) a 3-month total rainfall forecast made on the first day of every month; and (ii) a downscaled daily time series of rainfall, maximum and minimum temperatures and solar radiation, suitable to be used with a crop simulation model^35^.

### APSIM modelling

The open source code validated APSIM sorghum model^20^ was used to simulate a comprehensive factorial combination of hybrid characteristics (G), management factors and site-soil conditions across four locations in Queensland and New South Wales, Australia. APSIM sorghum was developed and parameterized using local field experimentation and cultivars, details on model equations and validation results are provided with the supplementary material, and can be also accessed at http://www.apsim.info/APSIM.Validation/Main.aspx

Simulations for the period 1981–2015 using observed station data (n = 1) and the downscaled daily time series forecast (n = 99) from POAMA were conducted for monthly sowing windows between September and January; on three different soil types per location (Table 2); and four values of initial soil water (ISW) at the time of sowing (0.2, 0.4, 0.6, and 0.8 v/v). Agronomic management factors included two row configurations (solid 1 m rows, and single skip)^36^, four levels of plant density (3.5, 5, 6.5, 8 pl m^−2^), and three levels of nitrogen fertilization (50, 100 and 150 kg N ha^−1^). Genotype characteristics included three levels of maturity (early, medium and late), and three levels of tillering (no tillering, medium and high tillering) as in^7^. The model was run over the 1981–2015 (34 years) hindcast series, resulting in a total number of GxExM combinations of 176,256,000 crop year simulations.

### Measures of skill

The skill of both forecasting systems was calculated on both 3-month gridded rainfall (GPCC) and simulated sorghum yields. From the rainfall forecast distributions, probabilities of above median, tercile 1 and 3 rainfall, and a binary outcome i.e. correct or incorrect, for each forecast were calculated. Measures of skill included the reliability diagram^37^, the Brier Skill Score (BSS, equations (S3 and S4))^38^, percent consistent rates, and measures of shift in the mean i.e. absolute mean deviation (AMD, equation (S5)), and dispersion i.e. the variance ratio (VR, equation (S6)) of the sample variances^18,39^.

Percent consistent compares how often the forecast favored a particular outcome and how often that outcome was realized^15^. Reliability diagrams indicate errors associated with the issued probabilities. In the reliability plots the y-axis is the relative observed frequency and the x-axis is the forecast probability. The solid line in the reliability plots indicates perfect reliability. The reliability diagram is accompanied by a histogram which indicates the sample size in each forecast probability bin. The histogram can be used to indicate possible sampling issues associated with each bin (e.g. too few samples for a robust result), as well as the sharpness of the forecast system. If all the forecasts fell in the model climatology probability bin, then the system would have no sharpness (sharpness is the tendency to forecast extreme values). Sharp forecasts are useful for decision making, assuming they are reliable. The Brier Skill Score indicates the proportional improvement of the probabilistic forecasts from a given system over using climatological forecasts. All statistical analyses were calculated using the R software^38^.

### Value of linking APSIM and POAMA-2 to inform crop designs (GxM)

The value of using POAMA-2 to inform more profitable and resilient crop designs was derived for the subset of GxExM combinations producing positive BSS values for forecasts of above/below median sorghum yields. A search algorithm was developed that included the following steps (i) a matrix of average profits and down side risk for best farmers’ practice and for a factorial combination of GxM (hybrid maturity and tillering type, plant density, row configuration and fertilization rate) was created; (ii) GxM combinations having values of BSS higher than zero (skill) for predicted profits were kept; (iii) For each year in the hindcast the GxM alternative showing the highest profit were then selected; (iv) Value was then calculated in terms of changes in average profits and down side risk i.e. likelihood of a profit lower than 600 AU$ ha^−1^, between best farmers’ practice, i.e. a static though locally recommended best agronomic management, and the selected GxM combination predicted using the POAMA-2 forecast for each year in the hindcast series, 1981–2015 (Value_optS_); and relative to the simulated most frequent optimum static GxM strategy (Value_optSCF_) (Table S2). In this analysis Value_optS_ represents both gains from improved crop design and the use of climate information, while Value_optSCF_ represents the actual value of the new climate information. As a reference point, value was also calculated for the hypothetical situation that the future climate was known i.e. perfect knowledge (Value_PK_). Value_PK_ was calculated as the difference in profit between the optimized crop design and farmers’ practice using observed climatology. To account for opportunity and fixed costs, a threshold value of 600 AU$ ha^−1^ to calculate down side risk was chosen after consultation with farmers across the locations in the study. Profits were calculated for median sorghum prices over the last 10 years (2007 to 2016) i.e. 254 AU$ t^−1^. Variable costs included insurance i.e. 1% of the gross income, while the fertilizer cost was 30 cents kg^−1^ urea (http://agmargins.net.au). Variable costs for failed crops were then 161 AU$ ha^−1^ (excluding harvest costs), and 211 AU$ ha^−1^ for harvested crops.

### Data and model availability

APSIM is an open source cropping systems model available at www.apsim.info.

## Electronic supplementary material

Supplementary information

## References

[1] Keating, B. A., Herrero, M., Carberry, P. S., Gardner, J. & Cole, M. B. Food wedges_ Framing the global food demand and supply challenge towards 2050. *Global Food Security* 1–8 10.1016/j.gfs.2014.08.004 (2014).

[2] Godfray HCJ, Garnett T (2014). Food security and sustainable intensification. Philosophical Transactions of the Royal Society B: Biological Sciences.

[3] Garnett T (2013). Sustainable Intensification in Agriculture: Premises and Policies. Science.

[4] Lobell DB, Cassman KG, Field CB (2009). Crop Yield Gaps: Their Importance, Magnitudes, and Causes. Annu. Rev. Environ. Resourc..

[5] van Ittersum MK (2013). Yield gap analysis with local to global relevance - A review. Field Crops Research.

[6] Chauhan YS, Solomon KF, Rodriguez D (2013). Characterization of north-eastern Australian environments using APSIM for increasing dryland maize production. Field Crops Research.

[7] Hammer GL (2014). Crop design for specific adaptation in variable dryland production environments. Crop Pasture Sci..

[8] Borrell AK (2014). Drought adaptation of stay-green sorghum is associated with canopy development, leaf anatomy, root growth, and water uptake. Journal of Experimental Botany.

[9] Potgieter AB (2016). Yield trends under varying environmental conditions for sorghum and wheat across Australia. Agricultural and Forest Meteorology.

[10] Monjardino M, McBeath TM, Brennan L, Llewellyn RS (2013). Are farmers in low-rainfall cropping regions under-fertilising with nitrogen? A risk analysis. Agricultural Systems.

[11] Meza FJ, Hansen JW, Osgood D (2008). Economic Value of Seasonal Climate Forecasts for Agriculture: Review of Ex-Ante Assessments and Recommendations for FutureResearch. J. Appl. Meteor. Climatol..

[12] Hansen J, Baethgen W, Osgood D, Ceccato P, Ngugi RK (2007). Innovations in Climate Risk Management: Protecting and Building Rural Livelihoods in a Variable and Changing Climate. Journal of Semi-Arid Tropical Agricultural Research.

[13] Bauer P, Thorpe A, Brunet G (2015). The quiet revolution of numerical weather prediction. Nature.

[14] Risbey JS, Pook MJ, McIntosh PC, Wheeler MC, Hendon HH (2009). On the Remote Drivers of Rainfall Variability in Australia. Mon. Wea. Rev..

[15] Charles, A. N., Duell, R. E. & Wang, X. *Seasonal Forecasting for Australia using a Dynamical Model: Improvements in Forecast Skill over the Operational Statistical Model*. *Australian Meteorological and Oceanographic Journal* **65**, 3–4 (AUSTRALIAN …, 2015).

[16] Hansen J (2004). Linking dynamic seasonal climate forecasts with crop simulation for maize yield prediction in semi-arid Kenya. Agricultural and Forest Meteorology.

[17] Hammer GL, Holzworth D, Stone R (1996). The value of skill in seasonal climate forecasting to wheat crop management in a region with high climatic variability. Aust J Agric Res.

[18] Potgieter AB, Everingham YL, Hammer GL (2003). On measuring quality of a probabilistic commodity forecast for a system that incorporates seasonal climate forecasts. Int. J. Climatol..

[19] Crane TA (2010). Forecast Skill and Farmers’ Skills: Seasonal Climate Forecasts and Agricultural Risk Management in the Southeastern United States. Wea. Climate Soc..

[20] Hammer GL (2010). Adapting APSIM to model the physiology and genetics of complex adaptive traits in field crops. Journal of Experimental Botany.

[21] Holzworth DP (2014). APSIM - Evolution towards a new generation of agricultural systems simulation. Environmental Modelling & Software.

[22] van Dijk AIJM (2013). The Millennium Drought in southeast Australia (2001–2009): Natural and human causes and implications for water resources, ecosystems, economy, and society. Water Resour. Res..

[23] Hammer GL (2001). Advances in application of climate prediction in agriculture. Agricultural Systems.

[24] Hayman P, Crean J, Mullen J, Parton K (2007). How do probabilistic seasonal climate forecasts compare with other innovations that Australian farmers are encouraged to adopt?. Aust. J. Agric. Res..

[25] Hudson D, Marshall AG, Yin Y, Alves O, Hendon HH (2013). Improving Intraseasonal Prediction with a New Ensemble Generation Strategy. Mon. Wea. Rev..

[26] Stone R, Hammer GL, Marcusen T (1996). Prediction of global rainfall probabilities using phases of the Southern Osclillation Index. Nature.

[27] Zhao M, Hendon HH, Alves O, Liu G, Wang G (2016). Weakened Eastern Pacific El Niño Predictability in the Early Twenty-First Century. J. Climate.

[28] Drosdowsky W, Chambers L (2001). Near-global sea surface temperature anomalies as predictors of Australian seaosonal rainfall. J. Climate.

[29] Power SB, Kociuba G (2010). The impact of global warming on the Southern Oscillation Index. Clim Dyn.

[30] Kirtman BP, Schopf PS (1998). Decadal variability in ENSO predictability and prediction. J. Climate.

[31] Wang E, Cresswell H, Xu J, Jiang Q (2009). Capacity of soils to buffer impact of climate variability and value of seasonal forecasts. Agricultural and Forest Meteorology.

[32] Wilks DS (2010). Sampling distributions of the Brier score and Brier skill score under serial dependence. Quarterly Journal of the Royal Meteorological Society.

[33] Meinke H, Stone R (2005). Seasonal and inter-annual climate forecasting: The new tool for increasing preparedness to climate variability and change in agricultural planning operations. Climatic Change.

[34] Goddard L (2001). Current approaches to seasonal to interannual climate predictions. Int. J. Climatol..

[35] McIntosh, P. C. & Brown, J. Calibration and bias correction of seasonal climate forecasts for use in agricultural models. 1–28, 10.4225/08/5910c03c4d437 (2017).

[36] Whish J (2005). Modelling the effects of row configuration on sorghum yield reliability in north-eastern Australia. Aust. J. Agric. Res..

[37] Bröcker J, Smith LA (2007). Increasing the Reliability of Reliability Diagrams. Wea. Forecasting.

[38] Murphy AH (1986). A new decomposition of the Brier score: Formulation and interpretation. Mon. Wea. Rev..

[39] Fawcett F, Stone R (2010). A comparison of two seasonal rainfall forecasting systems for Australia. Australian Meteorological and Oceanographic Journal.

