# Supplementary material for: Predicting optimum crop designs using crop models and seasonal climate forecasts
Source: Sci Rep. 2018 Feb 2;8:2231. doi: 10.1038/s41598-018-20628-2 (PMC5797250; doi:10.1038/s41598-018-20628-2)
Supplement: Supplementary file 1 — Supplementary information [file 41598_2018_20628_MOESM1_ESM.docx]

**Predicting optimum crop designs using crop models and seasonal climate forecasts**

Rodriguez D^1*^, deVoil P^1^, Hudson, D^2^, Brown J.N.^3^, Hayman P^4^, Marrou H^5^, Meinke H^6^

^1^ Queensland Alliance for Agriculture and Food Innovation (QAAFI), The University of Queensland, Australia

^2^ Bureau of Meteorology, Melbourne, Australia

^3^ CSIRO Agriculture and Food, 15 College Rd, Sandy Bay 7005, Australia

^4^South Australian Research and Development Institute, Adelaide, Australia

^5^SupAgro, 2 place Viala, 34060 Montpellier cedex 02, France

^6^University of Tasmania, and Tasmanian Institute of Agriculture, Hobart, Australia

**Measures of skill, shift and dispersion**

The Brier score (Murphy, 1986) represents the mean squared error of the probabilistic forecast.

$$BS=\frac{1}{n}\sum_{k=1}^{n} {(F_{k}-O_{k})}^{2} (S3)$$

In equation (S3) O_k_ is assigned a value of unity if the forecast event occurred e.g. exceed the median, and it is assigned a value of zero if the forecast did not eventuate. The average squared difference between the forecast probability F_k_ and the subsequent binary observation O_k_ is the Brier score over the evaluated period (n).

The BSS instead, measures the improvement of the probabilistic forecast relative to a reference forecast i.e. long-term climatology, thus taking climatological frequency into account. A BSS = 1 indicates a perfect score.

$$BSS=1- \frac{BS- \mathrm{BS}_{\mathrm{Reference}}}{\mathrm{BS}_{\mathrm{Reference}}} (S4)$$

The absolute mean deviation (AMD, equation (S5)) is calculated measuring the shift in the mean of the forecast (F_F_) distribution, relative to the reference (F_R_) distribution (Potgieter et al., 2003).

$$\mathrm{AMD}_{{(F}_{F}, F_{R})=|\bar{X}_{R}- \bar{X}_{F}|} (S5)$$

Where, $\bar{X}_{F}\mathrm{and}\bar{X}_{R}$ are the sample means of the forecasted and reference distributions F_F_ and F_R_, respectively.

The ratio of the sample variances is the variance ratio (VR, equation (S6)), and represents a measure of distribution dispersion (Potgieter et al., 2003).

$$\mathrm{VR}_{{(F}_{A}, F_{R}) = \frac{S_{F}^{2}}{S_{R}^{2}}} (S6)$$

Where $S_{F}^{2}$ and $S_{R}^{2}$ are the sample variances of the forecasted and reference distributions.

Murphy, A. H. A new decomposition of the Brier score: Formulation and interpretation. Mon. Wea. Rev. 114, 2671–2673 (1986).

Potgieter, A. B., Everingham, Y. L. & Hammer, G. L. On measuring quality of a probabilistic commodity forecast for a system that incorporates seasonal climate forecasts. Int. J. Climatol. 23, 1195–1210 (2003).

**Climate forecasts**
Crop yields are dependent upon a combination of weather factors – rainfall, radiation and temperature. In dryland cropping, rainfall is typically the limiting factor to growth over temperature and radiation. As a first test of crop forecasts the skill of a leading seasonal climate forecast model POAMA-2 (Hudson et al. 2013) for rainfall is contrasted with the commonly used statistical method the Southern Oscillation Index (SOI) phase system (Stone et al. 1996).

The SOI phase system is an analogue-year-based predictor. The SOI phase at the time of the forecast is determined being either consistently negative, consistently positive, rapidly falling, rapidly rising or consistently near zero and the years with similar phases between (1901 and 2013) are chosen to provide a prediction for this year. To run APSIM the daily records of observed rainfall, radiation, and minimum and maximum temperatures were taken from these analogue years.

In contrast, the POAMA-2 model is a dynamical coupled climate model initialised with data assimilated observations and has a nine-month forecast period. At each start time a 33-member ensemble is run to sample uncertainty and the ensemble mean is taken to be the representative forecast. The output of rainfall from the model is daily on a 240 km grid. To deal with this scale mismatch and possible model biases, the output is downscaled and calibrated to the nearest weather station location using a quantile mapping approach (McIntosh and Brown, 2017). The downscaled daily outputs of rainfall, radiation and maximum and minimum temperatures are then available from the POAMA-2 as inputs into the APSIM model.

Hudson, D., Marshall, A. G., Yin, Y., Alves, O. & Hendon, H. H. Improving Intraseasonal Prediction with a New Ensemble Generation Strategy. *Mon. Wea. Rev.* **141,** 4429–4449 (2013).

McIntosh, P. C. & Brown, J. Calibration and bias correction of seasonal climate forecasts for use in agricultural models. 1–28 (2017). doi: <https://doi.org/10.4225/08/5910c03c4d437>

Stone, R.C., Hammer, G.L and Marcussen, T. (1996) Prediction of global rainfall probabilities using phases of the Southern Oscillation Index. Nature, 384, 252-255.

**APSIM sorghum description and validation**

The Agricultural Production Systems sIMulator (APSIM) is internationally recognised as a highly advanced simulator of agricultural systems. APSIM is available either for non-commercial or commercial purposes, through the APSIM Community Source Framework. Through this framework, the intent is to develop a high-quality and enduring agricultural production systems modelling platform with global reach. APSIM may be used for applications in agricultural systems, for developing science-based enhancements to the modelling capability, or a combination of both.

APSIM contains a suite of modules which enable the simulation of systems that cover a range of plant, animal, soil, climate and management interactions. APSIM is undergoing continual development, with new capability added to regular releases of official versions. Its development and maintenance is underpinned by rigorous science and software engineering standards.  The source code of APSIM can be accessed from:

<http://apsrunet.apsim.info/websvn/listing.php?repname=apsim&path=%2Ftrunk%2F&template=Elegant>

A detailed description of APSIM sorghum is available from:

<http://www.apsim.info/Documentation/Model,CropandSoil/CropModuleDocumentation/Sorghum.aspx>

Results from model validation for a range of output variables e.g. biomass at flowering and maturity, leaf area at flowering, grain size, grain number and final yield are available from:

<http://www.apsim.info/APSIM.Validation/Main.aspx>

**Supplementary figures and tables**

Figure S1. Brier Skill Scores for forecasts of tercile 1 for three-monthly rainfall forecasted using POAMA2 and the SOI phase system for: all the locations (All), and for Capella (Cap), Dalby (Dalb), Goondiwindi (Gnd) in Queensland, and Moree (Mre) in New South Wales Australia.

Figure S2. Brier Skill Scores for forecasts of tercile 1 for three-monthly rainfall forecasted using POAMA2 and the SOI phase system for: all the locations (All), and for Capella (Cap), Dalby (Dalb), Goondiwindi (Gnd) in Queensland, and Moree (Mre) in New South Wales Australia.

Figure S3. Relationship between the variance ratio i.e. a measure of dispersion, and the absolute mean deviation (mm) i.e. a measure of shift, for monthly forecasts of three-monthly above median rainfall using POAMA2 and the SOI phase system for locations in the study together i.e. Capella, Dalby, and Goondiwindi in Queensland, and Moree in New South Wales Australia.

| Table S1. Analysis of variance for variables related to genotype characteristics (G), management practices (M) and location (E), for all the locations together (All sites); and Capella, Dalby, Goondiwindi, and Moree individually, for the GxMxE combinations showing Brier Skill Scores in the upper 75% of the values. | | | | | | |
| --- | --- | --- | --- | --- | --- | --- |
| **All sites** | **df** | **SS** | **MSS** | **F value** | **Pr(>F)** | **Significance** |
| Location | 3 | 15.9 | 5.3 | 1098 | < 2e-16 | *** |
| Sowing window | 4 | 169 | 42.2 | 8730 | < 2e-16 | *** |
| Soil type | 2 | 0.14 | 0.07 | 14 | < 5e-07 | *** |
| Plant available water | 3 | 1.15 | 0.38 | 79 | < 2e-16 | *** |
| Hybrid maturity | 2 | 0.34 | 0.17 | 35 | < 6e-16 | *** |
| Tillering type | 2 | 0.28 | 0.14 | 29 | < 2e-13 | *** |
| Density | 3 | 0.49 | 0.16 | 33 | < 2e-16 | *** |
| Row configuration | 1 | 0.06 | 0.06 | 12 | <0.001 | *** |
| Nitrogen fertilisation | 2 | 0.28 | 0.14 | 29 | <2e-13 | *** |
| Residuals | 51808 | 250 |  |  |  |  |
| **Capella** | **df** | **SS** | **MSS** | **F value** | **Pr(>F)** | **Significance** |
| Sowing window | 4 | 30 | 7.6 | 2301 | < 2e-16 | *** |
| Soil type | 2 | 0.65 | 0.3 | 97 | < 2e-16 | *** |
| Plant available water | 3 | 1.3 | 0.7 | 200 | < 2e-16 | *** |
| Hybrid maturity | 2 | 0.2 | 0.1 | 33 | < 4e-15 | *** |
| Tillering type | 2 | 0.6 | 0.3 | 87 | < 2e-16 | *** |
| Density | 3 | 0.4 | 0.1 | 40 | < 2e-16 | *** |
| Row configuration | 1 | 0.03 | 0.02 | 7.5 | <0.01 | ** |
| Nitrogen fertilisation | 2 | 1.3 | 0.6 | 200 | < 2e-16 | *** |
| Residuals | 12940 | 43 | 0.003 |  |  |  |
| **Dalby** | **df** | **SS** | **MSS** | **F value** | **Pr(>F)** | **Significance** |
| Sowing window | 4 | 41 | 10 | 2628 | < 2e-16 | *** |
| Soil type | 2 | 0.01 | 0.005 | 1.2 | 0.28 | ns |
| Plant available water | 3 | 1.8 | 0.6 | 157 | < 2e-16 | *** |
| Hybrid maturity | 2 | 0.9 | 0.4 | 114 | < 2e-16 | *** |
| Tillering type | 2 | 0.04 | 0.02 | 5 | <0.01 | ** |
| Density | 3 | 0.01 | 0.002 | 0.4 | 0.7 | ns |
| Row configuration | 1 | 0.2 | 0.2 | 50 | < 1e-12 | *** |
| Nitrogen fertilisation | 2 | 1.5 | 0.8 | 200 | < 2e-16 | *** |
| Residuals | 12940 | 50 | 0.004 |  |  |  |
| **Goondiwindi** | **df** | **SS** | **MSS** | **F value** | **Pr(>F)** | **Significance** |
| Sowing window | 4 | 110 | 27 | 6082 | < 2e-16 | *** |
| Soil type | 2 | 0.91 | 0.45 | 100 | < 2e-16 | *** |
| Plant available water | 3 | 0.99 | 0.33 | 72 | < 2e-16 | *** |
| Hybrid maturity | 2 | 0.22 | 0.11 | 24 | <2e-11 | *** |
| Tillering type | 2 | 0.03 | 0.14 | 3.1 | 0.04 | * |
| Density | 3 | 0.23 | 0.07 | 17 | <4.e-11 | *** |
| Row configuration | 1 | 0 | 0.001 | 0.3 | 0.6 | ns |
| Nitrogen fertilisation | 2 | 0 | 0.002 | 0.5 | 0.6 | ns |
| Residuals | 12931 | 58 | 0.005 |  |  |  |
| **Moree** | **df** | **SS** | **MSS** | **F value** | **Pr(>F)** | **Significance** |
| Sowing window | 4 | 110 | 27.5 | 6082 | < 2e-16 | *** |
| Soil type | 2 | 0.91 | 0.45 | 100 | < 2e-16 | *** |
| Plant available water | 3 | 0.99 | 0.33 | 72 | < 2e-16 | *** |
| Hybrid maturity | 2 | 0.22 | 0.11 | 24 | <2e-11 | *** |
| Tillering type | 2 | 0.03 | 0.01 | 3 | 0.04 | * |
| Density | 3 | 0.23 | 0.07 | 17 | <4e-11 | *** |
| Row configuration | 1 | 0 | 0.001 | 0.27 | 0.6 | ns |
| Nitrogen fertilisation | 2 | 0 | 0.002 | 0.4 | 0.6 | ns |
| Residuals | 12931 | 58 | 0.005 |  |  |  |
| Degrees of freedom (df), Sum of squares (SS), Mean sum of squares (MSS) | | | | | | |
| Significance codes: ***= 0; **= 0.001; ** = 0.01; and * = 0.05; ns = not significant | | | | | | |

| Table S2. Location, soil type, farmers’ most common hybrid (G) by management (M) combination, and most frequent POAMA-2 optimum GxM combination together with their mean profit, down side risks and changes with respect to farmers’ GxM. The last column indicates the proportion of years the strategy was realised. | | | | | | | |
| --- | --- | --- | --- | --- | --- | --- | --- |
| Location | Farmers' GxM | Soil type (PAWC) | POAMA-2 optimum GXM | Profit ($ ha^-1^) | % change | Down side risk (%) | % years realised |
| Capella, Qld | January sowing, on 60% ISW, early maturity and medium tillering hybrid, sown in solid configuration at 5 pl m^-2^, and fertilised with 50 kg N ha^-1^ | High | January sowing, on 60% ISW, early maturity and medium tillering hybrid, sown in solid configuration at 8 pl m^-2^ and fertilised with 100 kg N ha^-1^ | 1217 | 10 | 0 | 41 |
|  |  | Medium | January sowing, on 60% ISW, early maturity and medium tillering hybrid, sown in solid configuration at 8 pl m^-2^ and fertilised with 100 kg N ha^-1^ | 762 | 2 | 0 | 50 |
|  |  | Low | January sowing, on 60% ISW, early maturity and high tillering hybrid, sown in solid configuration at 6.5 pl m^-2^ and fertilised with 100 kg N ha^-1^ | 670 | 23 | 36 | 41 |
| Dalby, Qld | October sowing, on 60% ISW, medium maturity and medium tillering hybrid, sown in solid configuration at 5 pl m^-2^, and fertilised with 50 kg N ha^-1^ | High | October sowing, on 60% ISW, early maturity and medium tillering hybrid, sown in solid configuration at 6.5 pl m^-2^, and fertilised with 100 kg N ha^-1^ | 1312 | 16 | 0 | 26 |
|  |  | Medium | October sowing, on 60% ISW, early maturity and medium tillering hybrid, sown in solid configuration at 8 pl m^-2^, and fertilised with 100 kg N ha^-1^ | 1357 | 29 | 0 | 26 |
|  |  | Low | October sowing, on 60% ISW, late maturity and no tillering hybrid, sown in solid configuration at 8 pl m^-2^, and fertilised with 100 kg N ha^-1^ | 964 | 21 | 0 | 26 |
| Goondiwindi, Qld | September sowing, on 60% ISW, medium maturity and medium tillering hybrid, sown in solid configuration at 5 pl m^-2^, and fertilised with 50 kg N ha^-1^ | High | September sowing, on 60% ISW, early maturity and high tillering hybrid, sown in solid configuration at 3.5 pl m^-2^, and fertilised with 100 kg N ha^-1^ | 1036 | 20 | 0 | 53 |
|  |  | Medium | September sowing, on 60% ISW, early maturity and no tillering hybrid, sown in solid configuration at 6.5 pl m^-2^, and fertilised with 100 kg N ha^-1^ | 865 | 3 | 0 | 29 |
|  |  | Low | September sowing, on 60% ISW, early maturity and no tillering hybrid, sown in solid configuration at 8 pl m^-2^, and fertilised with 100 kg N ha^-1^ | 832 | 23 | 0 | 50 |
| Moree, NSW | September sowing, on 60% ISW, medium maturity and medium tillering, sown in solid configuration at 5 pl m^-2^, and fertilised with 50 kg N ha^-1^ | High | September sowing, on 60% ISW, early maturity and medium tillering hybrid, sown in solid configuration at 5 pl m^-2^, and fertilised with 100 kg N ha^-1^ | 1222 | 19 | 0 | 29 |
|  |  | Medium | September sowing, on 60% ISW, early maturity and no tillering hybrid, sown in solid configuration at 8 pl m^-2^, and fertilised with 100 kg N ha^-1^ | 871 | 7 | 0 | 12 |
|  |  | Low | September sowing, on 60% ISW, early maturity and high tillering hybrid, sown in solid configuration at 8 pl m^-2^, and fertilised with 100 kg N ha^-1^ | 329 | -11 | 100 | 35 |
|  | | | | | | | |
